# Buffering of cytosolic calcium plays a neuroprotective role by preserving the autophagy-lysosome pathway during MPP^+^-induced neuronal death

**DOI:** 10.1038/s41420-019-0210-6

**Published:** 2019-08-19

**Authors:** Shinae Jung, Yuhyun Chung, Yunsoo Lee, Yangsin Lee, Jin Won Cho, Eun-Joo Shin, Hyoung-Chun Kim, Young J. Oh

**Affiliations:** 1 Department of Systems Biology, Yonsei University College of Life Science and Biotechnology, Seoul, 03722 Korea; 2 Glycosylation Network Research Center, Yonsei University, Seoul, 03722 Korea; 3 Neuropharmacology and Toxicology Program, College of Pharmacy, Kangwon National University, Chunchon, 24341 Korea

**Keywords:** Cell death in the nervous system, Neurological disorders

## Abstract

Parkinson’s disease (PD) is a chronic neurodegenerative disease with no cure. Calbindin, a Ca^2+^-buffering protein, has been suggested to have a neuroprotective effect in the brain tissues of PD patients and in experimental models of PD. However, the underlying mechanisms remain elusive. Here, we report that in 1-methyl-4-phenylpyridinium (MPP^+^)-induced culture models of PD, the buffering of cytosolic Ca^2+^ by calbindin-D28 overexpression or treatment with a chemical Ca^2+^ chelator reversed impaired autophagic flux, protecting cells against MPP^+^-mediated neurotoxicity. When cytosolic Ca^2+^ overload caused by MPP^+^ was ameliorated, the MPP^+^-induced accumulation of autophagosomes decreased and the autophagic flux significantly increased. In addition, the accumulation of damaged mitochondria and p62-positive ubiquitinated protein aggregates, following MPP^+^ intoxication, was alleviated by cytosolic Ca^2+^ buffering. We showed that MPP^+^ treatment suppressed autophagic degradation via raising the lysosomal pH and therefore reducing cytosolic Ca^2+^ elevation restored the lysosomal pH acidity and normal autophagic flux. These results support the notion that functional lysosomes are required for Ca^2+^-mediated cell protection against MPP^+^-mediated neurotoxicity. Thus, our data suggest a novel process in which the modulation of Ca^2+^ confers neuroprotection via the autophagy-lysosome pathway. This may have implications for the pathogenesis and future therapeutic targets of PD.

## Introduction

Parkinson’s disease (PD) is accompanied with a progressive loss of dopaminergic neurons in the substantia nigra pars compacta (SNpc) and dopamine depletion in the striatum^1,2^. Although the etiology of PD is unclear, accumulating evidence suggests that abnormal protein aggregation, mitochondrial dysfunction, and dysregulated Ca^2+^ homeostasis may be involved in the neurodegeneration observed in PD^3–6^. Recently, advances have been made in defining cell death modes associated with the pathogenesis of PD^7^. Role of apoptosis has been highlighted in studies using postmortem brains of PD patients and experimental models of PD that were generated by applying familial cases of gene mutations or treatment with neurotoxins^8^. Accumulating evidence implicates other cell death modes including necrosis, necroptosis, and parthanatos^9,10^. More recently, dysregulated autophagic pathway has been found in postmortem PD brains and in experimental models of PD^11–13^.

Because the intracellular catabolic process through which protein aggregates and damaged subcellular organelles can be degraded, autophagy is linked to PD pathogenesis^14–16^. Autophagy is a lysosomal degradation pathway, which is categorized into macroautophagy, chaperone-mediated autophagy, and microautophagy. Macroautophagy (hereafter referred to as autophagy) involves double-membrane vesicles called autophagosomes that sequester a portion of the cytosol. After autophagosomes are formed and subsequently fused with late endosomes and/or lysosomes, lysosomal hydrolases digest the internal cargo and the inner membrane of autophagosomes. Autophagy is essential for neuronal homeostasis and acts as a cytoprotective mechanism^17–19^. Consequently, defective autophagy leads to neurodegeneration^17,18^ and cumulative evidence has shown alterations in the autophagy-lysosome pathway in neurodegenerative disorders^15^. Notably, a tight link between autophagy and PD is supported by the finding that many PD-related genes are associated with the autophagy-lysosome pathway. For instance, parkin and PINK1 play major roles in mitophagy^20^. Similarly, α-synuclein is a substrate for chaperone-mediated autophagy, and pathogenic mutants in this gene interfere with that process^21^.

Because pathophysiological changes induced by neurotoxins were reminiscent of those seen in PD patients, these neurotoxins have been used for establishing experimental models of PD and investigating the potential pathophysiology associated with dopaminergic neurodegeneration^2^. Previously, we have suggested that these neurotoxins act on distinct cell death pathways^22–24^. For example, ROS play a crucial role in 6-hydroxydopamine (6-OHDA)-induced apoptosis, whereas treatment with 1-methyl-4-phenylpyridinium (MPP^+^) causes calcium-dependent cell death. We have determined using biochemical and ultrastructural criteria that these neurotoxins trigger autophagy^25,26^. More recently, we have indicated that a 6-OHDA triggers an excessive autophagic flux that precedes apoptosis^27^. Considering the findings that dysregulated autophagic flux may be linked to neuronal death, we questioned whether the buffering of cytosolic Ca^2+^ regulates the autophagy-lysosome pathway. Here, we demonstrated that MPP^+^-mediated overload of cytosolic Ca^2+^ was responsible for defective autophagy and resulted in cell death. We further identified that autophagic malfunction caused by MPP^+^ treatment was due to impaired autophagic degradation associated with lysosomal deficits. Accordingly, our data suggest that limiting the increase of cytosolic Ca^2+^ protects against MPP^+^-induced neuronal cell death via preserving the autophagy-lysosome pathway.

## Results

### Buffering of cytosolic Ca^2+^ attenuates MPP^+^-induced dopaminergic neuronal cell death

**Fig. 1:**
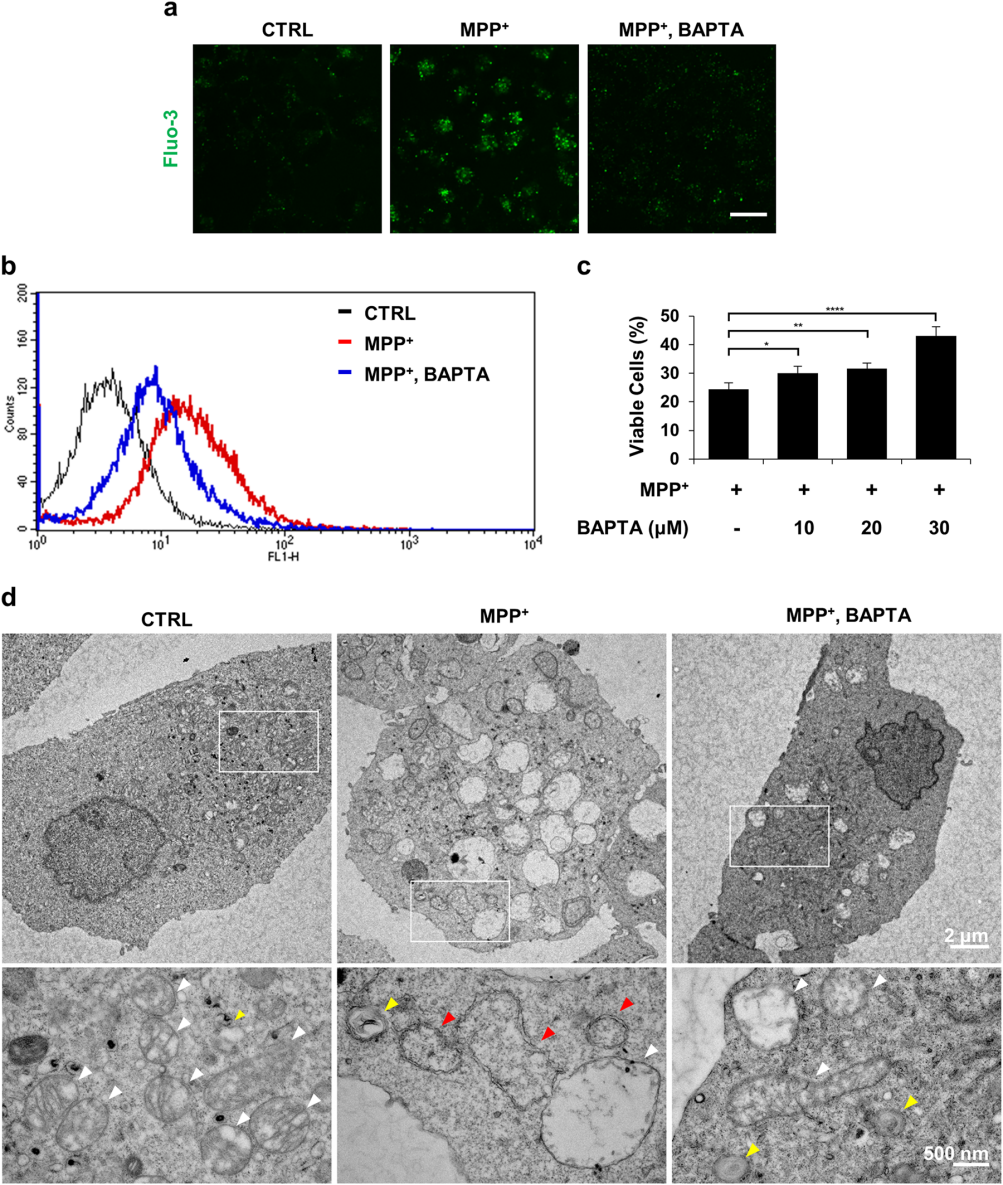
Buffering of cytosolic calcium attenuates MPP^+^-mediated toxicity in MN9D cells. **a**–**d** MN9D cells were treated with vehicle (control, CTRL) or 50 μM MPP^+^ in the presence or absence of 30 μM BAPTA-AM for 30 h. **a** MN9D cells were stained with 3 µM Fluo-3. A representative image of cells was taken using confocal microscopy after fixation. The scale bar represents 20 μm. **b** Cytosolic Ca^2+^ levels were assessed using flow cytometry. **c** MTT reduction assays were performed to assess cell viability that is expressed as a percentage of untreated control cells (100%). Bars represent the mean ± SEM of three independent experiments in triplicate. ^*^p < 0.05; ^**^p < 0.01; ^****^p < 0.0001. **d** Electron micrographs of MN9D cells were taken after drug treatment. Lower panels are magnified images from the boxed areas. Mitochondria (white arrowheads), lysosomes (yellow arrowheads) and autophagosomes (red arrowheads) are indicated

As previously demonstrated by us^28,29^, 1,2-Bis(2-aminophenoxy)ethane-N,N,N′,N′-tetraacetic acid tetrakis(acetoxymethyl ester)[BAPTA-AM] alleviated the elevation of Fluo-3-sensitive cytosolic Ca^2+^ in MN9D cells treated with MPP^+^ (Fig. 1a, b). MPP^+^-mediated cell death was inhibited by BAPTA-AM co-treatment (Fig. 1c). Transmission electron microscopy provided evidence that BAPTA-AM protects cells from MPP^+^ toxicity. MPP^+^ treatment resulted in the appearance of swollen mitochondria and autophagic vacuoles (Fig. 1d and Fig. S1). In contrast, co-treatment with BAPTA-AM maintained a pool of intact mitochondria. Similarly, fewer MPP^+^-induced autophagic vacuoles were detected in MN9D cells co-treated with BAPTA-AM (Fig. 1d). To verify the neuroprotective role of buffering of cytosolic Ca^2+^, MN9D cells were transfected with a vector containing calbindin-D28K (MN9D/CB) or empty control vector (MN9D/Neo; Fig. S2a). MPP^+^-induced elevation of cytosolic Ca^2+^ levels observed in MN9D/Neo cells was markedly limited in MN9D/CB #1 cells (Fig. S2b). In agreement with previous reports^30–32^, MPP^+^-induced cell death was suppressed in all three MN9D/CB cell lines (Fig. S2c), demonstrating that the buffering of MPP^+^-induced cytosolic Ca^2+^ surges inhibited dopaminergic neuronal death.

### MPP^+^-induced autophagosome accumulation is mediated by elevated cytosolic calcium

**Fig. 2:**
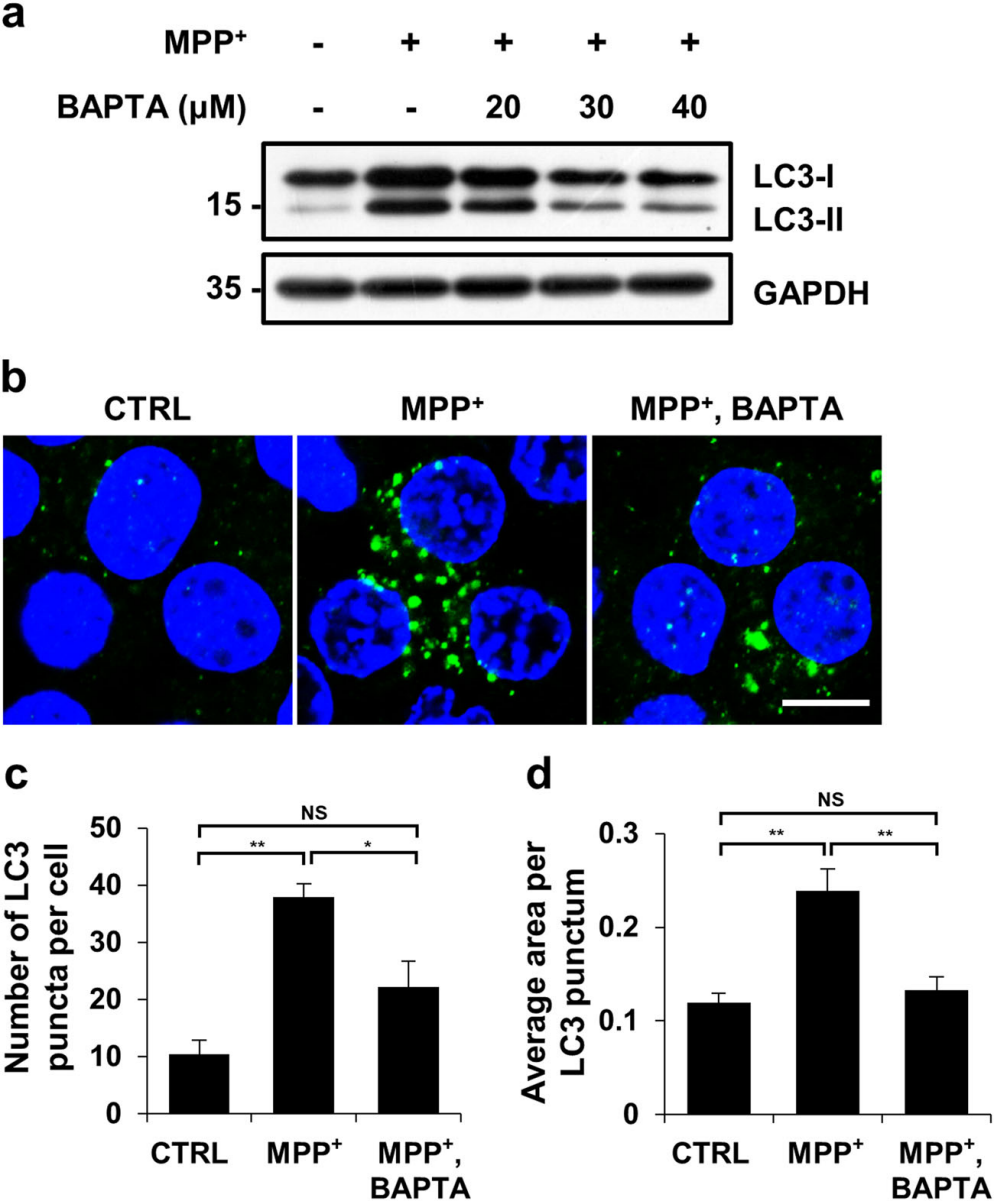
Reducing cytosolic calcium elevation suppresses the appearance of LC-3-positive autophagosomes caused by MPP^+^ treatment. MN9D cells were treated with 50 μM MPP^+^ in the presence or absence of the indicated concentrations of BAPTA-AM for 30 h. **a** Cell lysates were subjected to immunoblot analyses using the anti-LC3 antibody. Anti-GAPDH antibody was used as a loading control. **b**–**d** Immunocytochemical analyses were performed using an anti-LC3 antibody (green) and nuclei counterstaining with Hoechst 33258 (blue). **b** Cells were then examined using a confocal microscope. Merged images are provided. The scale bar represents 10 μm. The number of LC3 puncta per cell (**c**) and average puncta area of LC3 (**d**) were quantified using ImageJ after examining 90 cells per condition. The bar represents the mean ± SEM of three independent experiments. ^*^p < 0.05; ^**^p < 0.01; NS, not significant

Previous reports have demonstrated the existence of autophagosomes in postmortem PD brain and in experimental PD models^25,33–35^. Consistent with these findings, we observed double-membrane autophagosomes and autolysosomes in MPP^+^-treated cells, whereas these autophagic vacuoles were not easily detected in control cells or MPP^+^- and BAPTA-AM-co-treated cells (Fig. 1d and Fig. S1). To verify the correlation between cytosolic Ca^2+^ levels and autophagy during MPP^+^-induced neurodegeneration, titration experiments were carried out with increasing concentrations of BAPTA-AM, but a fixed dose of MPP^+^. The levels of LC3-II were increased by MPP^+^ treatment. However, the MPP^+^-induced increase in LC3-II levels were blocked by BAPTA-AM (Fig. 2a). Immunofluorescence studies revealed that the MPP^+^-induced increase in the number and average area of LC3 puncta was decreased in MN9D cells co-treated with MPP^+^ and BAPTA-AM (Fig. 2b–d). Immunoblotting and immunofluorescence analyses showed similar results for all three independent MN9D cell lines overexpressing calbindin-D28K (Fig. S3a–d). Taken together, data suggest that suppressing the MPP^+^-induced rise in cytosolic Ca^2+^ levels inhibited autophagosomal accumulation in MN9D cells.

### Buffering of MPP^+^-induced surge of cytosolic Ca^2+^ can normalize the autophagic flux

**Fig. 3:**
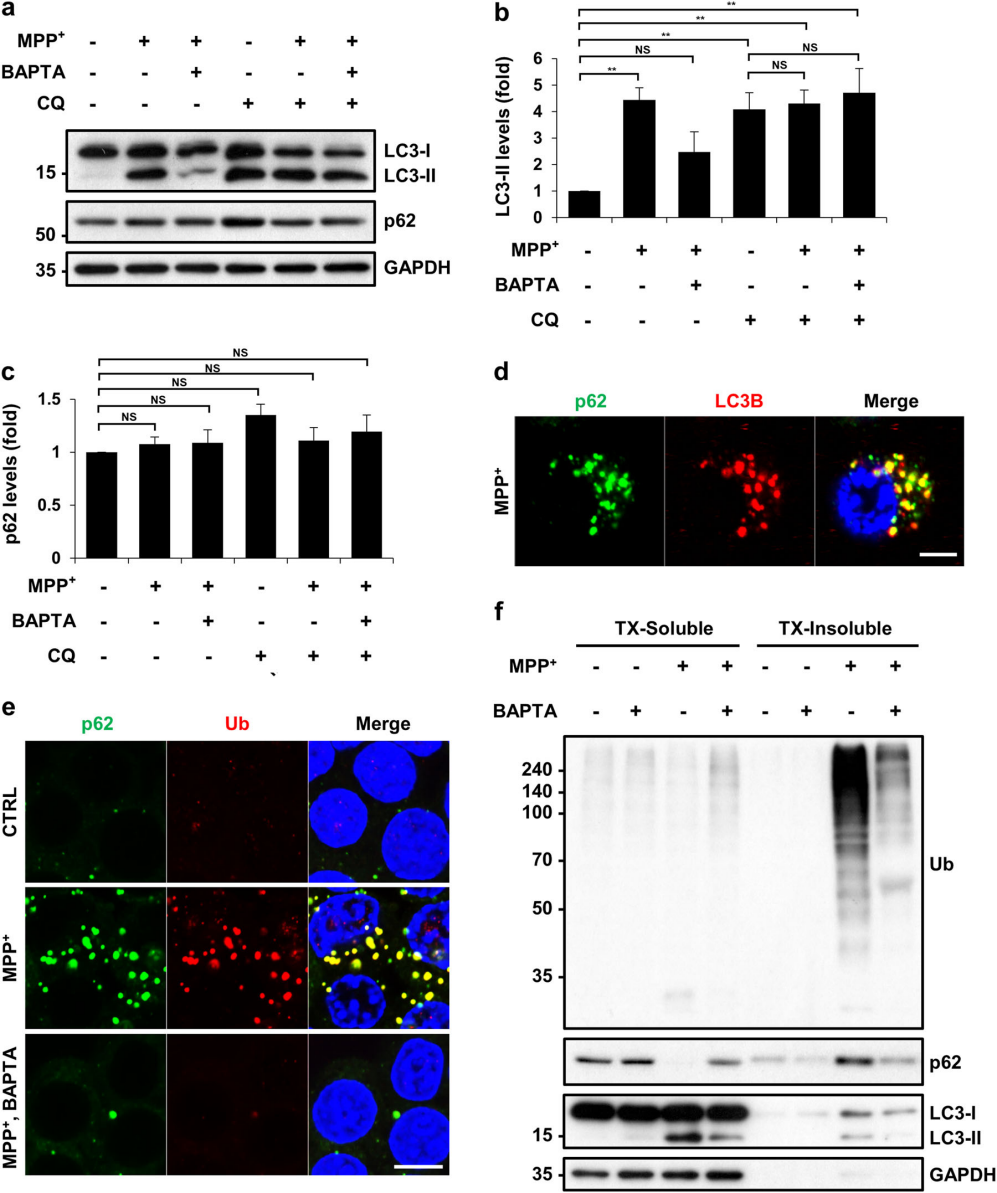
MPP^+^ blocks autophagic flux as determined by an elevated calcium-dependent accumulation of insoluble p62 and Ub-positive spots. MN9D cells were treated with 50 μM MPP^+^ in the presence or absence of 30 μM BAPTA-AM and/or 50 μM chloroquine (CQ) for 30 h. **a** Immunoblot analyses were performed using the anti-LC3 or anti-p62 antibody. **b**, **c** Quantification of LC3-II and p62 levels in each condition was performed after normalization to GAPDH loading control. Bars represent the mean ± SEM of three independent experiments. ^**^p < 0.01; NS, not significant. After MPP^+^ treatment alone (**d**) or in combination with BAPTA-AM (**e**), cells were analyzed for the immunofluorescent localization of p62 (green) and LC3B (red) or ubiquitin (Ub; red). Cells were then examined using a confocal microscope. Merged views are provided in the right panel. The scale bar represents (**d**) 5 μm and (**e**) 10 μm. **f** A representative immunoblot analyses of p62, ubiquitin, and LC3 in Triton X-100 (TX)-soluble or TX-insoluble fractions

To investigate the mechanism underlying autophagosomal accumulation following MPP^+^ treatment, we performed an autophagic flux assay using chloroquine (CQ), which inhibits lysosome-mediated degradation. Co-treatment with MPP^+^ and CQ did not further increase LC3-II levels compared with CQ treatment alone (Fig. 3a, lanes 4 and 5; Fig. 3b), suggesting that MPP^+^-induced accumulation of LC3-II was not due to an increase in the production of LC3-II. Instead, MPP^+^-induced accumulation of LC3-II appeared to be caused by impaired autophagic degradation, as there was no synergistic effect between CQ and MPP^+^. However, CQ increased LC3-II levels in cells co-treated with MPP^+^ and BAPTA-AM (Fig. 3a, lanes 3 and 6), indicating that there is no blockage in autophagic flux when cytosolic Ca^2+^ is buffered. We observed these autophagic events using endogenous autophagic marker LC3; however, another widely used autophagic substrate, p62, did not exhibit a similar trend—total cellular p62 levels were unaffected by MPP^+^ (Fig. 3a, c). Co-immunofluorescence studies showed that the cytosolic p62 puncta largely co-localized with LC3 puncta in MPP^+^-treated cells (Fig. 3d and Fig. S4). In MN9D cells co-treated with MPP^+^ and BAPTA-AM, p62 puncta were markedly reduced (Fig. 3e), suggesting that the levels of the insoluble form of p62 were reduced after BAPTA-AM co-treatment. p62 is an autophagic adaptor protein that can bind to LC3 and ubiquitin (Ub) simultaneously, thereby linking ubiquitinated targets to autophagosomes^36^. Indeed, immunofluorescence studies revealed that p62-positive and Ub-positive puncta co-localized in the cytosol in response to MPP^+^ treatment (Fig. 3e). These p62-positive Ub aggregates were not easily detected in cells co-treated with MPP^+^ and BAPTA-AM (Fig. 3e). To confirm this result, we carried out a 1% Triton X-100 soluble/insoluble fractionation experiment and found that MPP^+^ changed the solubility of p62 from the soluble to the insoluble fraction (Fig. 3f; compared with Fig. 3a, c). Consistent with the immunofluorescence data, immunoblot analyses indicated that ubiquitinated proteins accumulated in insoluble fractions following MPP^+^ treatment. Co-treatment with MPP^+^ and BAPTA-AM inhibited MPP^+^-induced accumulation of ubiquitinated proteins in the insoluble fraction. Taken together, these results indicate that MPP^+^ treatment impairs autophagic flux, causing the accumulation of p62 and ubiquitinated proteins in the undegraded autophagosomes; however, the buffering of cytosolic Ca^2+^ can normalize the autophagic flux.

To verify the correlation between increased cytosolic Ca^2+^ levels and impairment of autophagic flux, we used another cell death paradigm in which nigericin induces impairment of autophagic flux in MN9D cells^26^. Nigericin-induced surge of cytosolic Ca^2+^ was attenuated by BAPTA-AM (Fig. S5a). Nigericin-induced cell death was significantly suppressed by co-treatment with BAPTA-AM (Fig. S5b). Nigericin-induced accumulation of autophagic vacuoles was significantly inhibited in the presence of BAPTA-AM (Fig. S5c). Consistent with ultrastructural observations, immunoblot analyses showed that nigericin-induced increase in LC3-II levels were blocked by BAPTA-AM (Fig. S6a). Immunofluorescence studies indicated that co-treatment with nigericin and CQ did not further increase in the number and area of LC3 puncta compared with nigericin alone (Fig. S6b). However, CQ increased in the number and area of LC3 puncta in cells co-treated with nigericin and BAPTA-AM (Fig. S6b), supporting the notion that there is no blockage in autophagic flux when cytosolic Ca^2+^ is buffered.

### Normalization of cytosolic Ca^2+^ levels reverses the lysosomal pH deficits and impeded autophagic degradation caused by MPP^+^

**Fig. 4:**
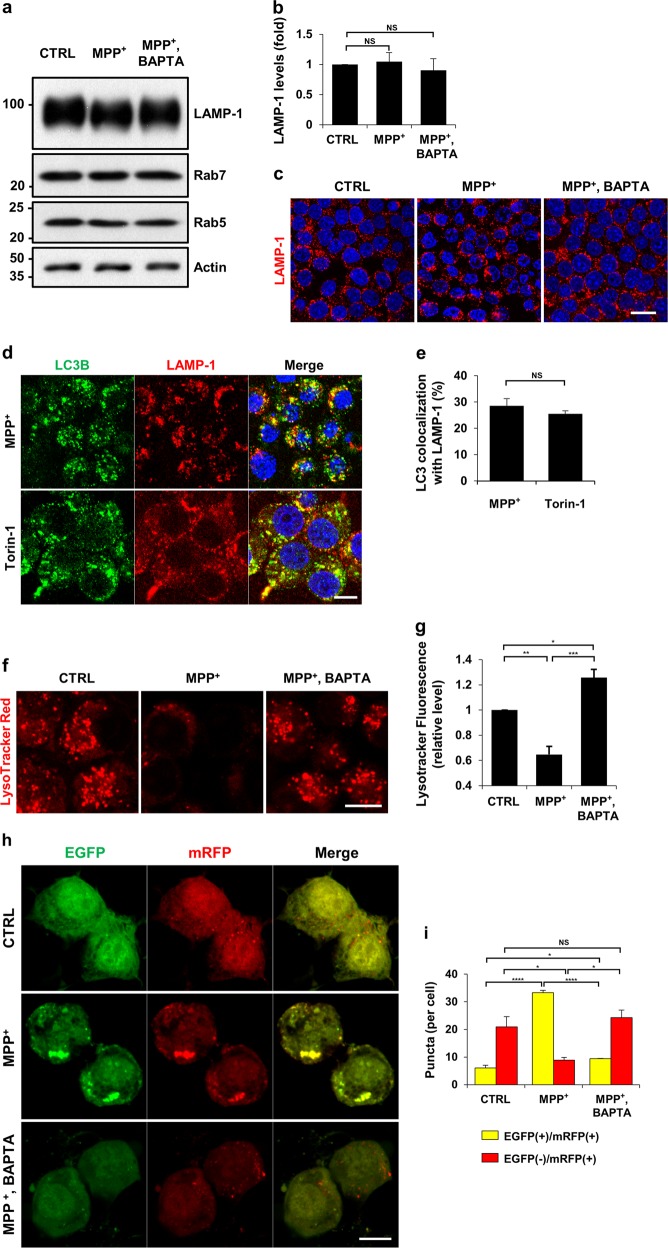
MPP^+^-induced impairment of autophagic degradation results from lysosomal malfunction in a calcium-dependent manner. **a**–**c** MN9D cells were treated with 50 μM MPP^+^ alone or in combination with 30 μM BAPTA-AM for 30 h. **a** Cell lysates were subjected to immunoblot analyses with the indicated antibodies. **b** Quantification of LAMP-1 expression was performed after normalization to actin loading control. Bars represent the mean ± SEM of three independent experiments. NS, not significant. **c** After drug treatment, cells were immunostained with anti-LAMP-1 antibody. Representative confocal images are provided. Scale bar represents 20 μm. **d**, **e** MN9D cells were incubated with 50 μM MPP^+^ for 30 h or 500 nM Torin-1 for 24 h and then immunostained using anti-LC3 and anti-LAMP-1 antibodies. The scale bar represents 10 μm. **e** Co-localization of LC3 with LAMP-1 in cells was quantified using ImageJ. Confocal images of at least 30 randomly selected cells from each of the three independent experiments were used for quantitation. Spots that were positive for both LC-3 and LAMP-1 were counted and expressed as a percentage of all LC3-positive spots (100%). Bars represent the mean ± SEM of three independent experiments. NS, not significant. **f**, **g** MN9D cells that were treated with 50 μM MPP^+^ alone or in combination with 30 μM BAPTA-AM for 30 h were stained with LysoTracker Red. Representative confocal images are provided. The scale bar represents 10 μm. **g** For quantitation, cells were subjected to flow cytometry. Data represent the fluorescence intensity relative to that of control cells (value = 1). Bar represents the mean ± SEM of three independent experiments. ^*^p < 0.05; ^**^p < 0.01; ^***^p < 0.001. **h** MN9D cells were transfected with mRFP-EGFP-tagged LC3B probe for 24 h and treated with 50 μM MPP^+^ alone or in combination with 30 μM BAPTA-AM for 30 h. After fixation, fluorescent images were acquired using confocal microscopy. The scale bar represents 10 μm. **i** Quantification of the number of yellow puncta (mRFP^+^-EGFP^+^-LC3B) and red puncta (mRFP^+^-EGFP^−^-LC3B) were performed using at least 50 cells per condition. Bar represents the mean ± SEM of three independent experiments. ^*^p < 0.05; ^****^p < 0.0001; NS, not significant

To explore the cause of impeded autophagic degradation by MPP^+^, we tested whether the number of lysosomes were affected by MPP^+^. The protein levels of LAMP-1 were not altered by treatment with MPP^+^ alone or in combination with BAPTA-AM (Fig. 4a, b). Immunofluorescence analyses showed that the cellular distribution of LAMP-1 was unaltered in all the groups (Fig. 4c). The levels of Rab5 and Rab7 remained unchanged regardless of drug treatment, indicating that MPP^+^ did not influence the distribution of the individual components of the endosomal pathway, including lysosomes. To study the effect of MPP^+^ treatment on autophagosome-lysosome fusion^37^, we analyzed the co-localization of LC3 with LAMP-1. Cells treated with Torin-1, an mTOR inhibitor exhibited a similar degree of co-localization between LC3 and LAMP-1 when compared with MN9D cells treated with MPP^+^ (Fig. 4d, e). Similarly, expression levels of other autophagosome-lysosome fusion markers, including syntaxin-17, p150Glued, and dynein intermediate chain was not altered regardless of MPP^+^ treatment (Fig. S7), demonstrating that MPP^+^ did not affect the fusion step between autophagosomes and lysosomes.

Optimal function of lysosomal hydrolases requires lysosomes to maintain a low internal pH^37^. To check whether the luminal pH of lysosomes was altered by MPP^+^ treatment, MN9D cells were stained with LysoTracker Red. In comparison with DMSO-treated cells, MPP^+^-treated cells lost fluorescence (Fig. 4f, g). Because there were no changes in the number of lysosomes, these results suggested that the luminal acidity was altered in MPP^+^-treated cells. Notably, co-treatment with MPP^+^ and BAPTA-AM did not elevate the lysosomal pH. To confirm MPP^+^-mediated elevation of lysosomal pH, we performed a fluorogenic activity assay for cathepsin B, a major lysosomal protease. Treatment with bafilomycin A1, an inhibitor of lysosomal acidification, showed that the elevation of lysosomal pH led to decreased activity of cathepsin B (Fig. S8). Along with an elevated lysosomal pH, cathepsin B activity was diminished by MPP^+^ treatment, which implied that autophagic degradation was defective. Buffering of cytosolic Ca^2+^ with BAPTA-AM restored normal cathepsin B activity in MPP^+^-treated cells. To provide additional evidence for lysosomal neutralization and resultant impaired autophagy following MPP^+^ treatment, we monitored autophagic flux by transiently expressing a tandom fluorescent-tagged LC3 probe (mRFP-EGFP-LC3), as described^38^. Under control conditions, yellow (red+/green+) puncta were barely seen, and approximately 20 % red (red+/green−) puncta were observed (Fig. 4h, i), suggesting that basal autophagy was operational, because most autophagosomes fused with lysosomes and were degraded due to low pH conditions. In contrast, in MPP^+^-treated cells, the numbers of red-only puncta were remarkably decreased and the numbers of yellow puncta increased. Because autophagosome-lysosome fusion is not impaired, these findings support the view that MPP^+^ interferes with lysosomal acidity, which prevents the quenching of GFP fluorescence. MN9D cells treated with MPP^+^ and BAPTA-AM showed a similar pattern to DMSO-treated control cells. Therefore, our data suggest that lysosomes fail to keep the luminal pH low in response to MPP^+^ treatment, which interrupts autophagic degradation; normalization of cytosolic Ca^2+^ levels reverses the lysosomal pH deficits caused by MPP^+^.

### Lowering cytosolic Ca^2+^ levels has a neuroprotective effect on MPP^+^-mediated cytotoxicity, independent of mTOR activity but dependent on lysosomal activity

**Fig. 5:**
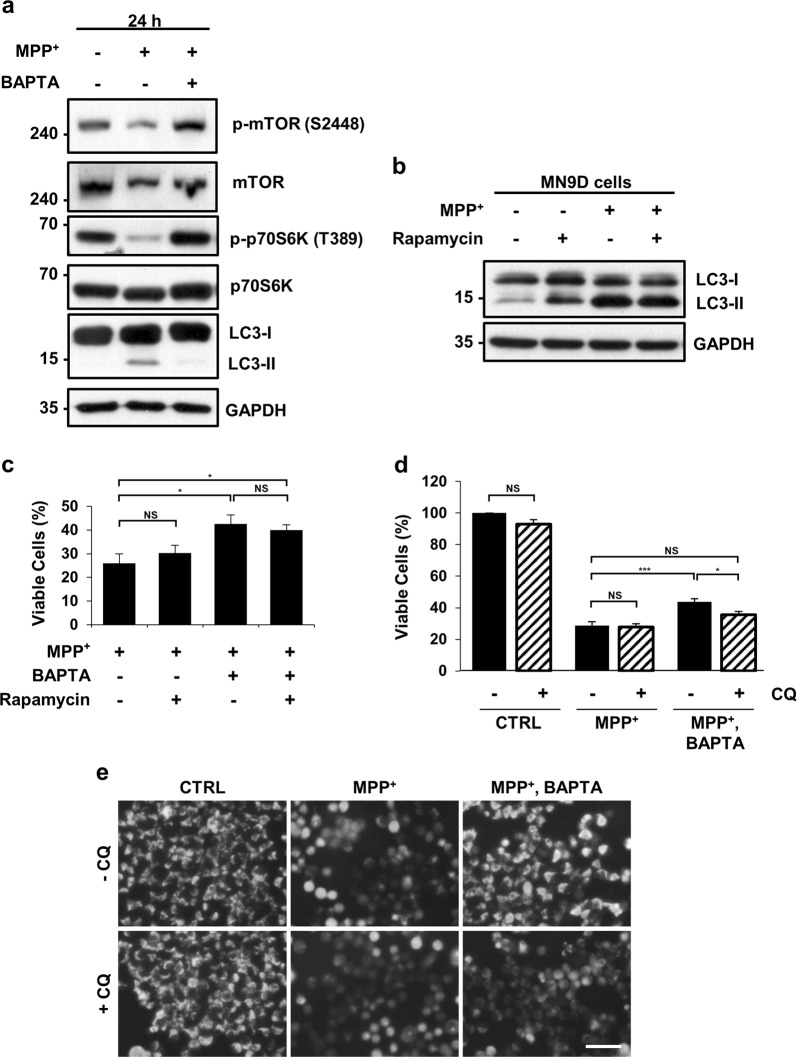
Lysosomal activity is essential for calcium-mediated cell protection against MPP^+^ toxicity. **a** MN9D cells were treated with 50 μM MPP^+^ in the presence or absence of 30 μM BAPTA-AM for 24 h. Cell lysates were subjected to immunoblot analyses using the indicated antibodies. Representative blots are provided. MN9D cells were treated with 50 μM MPP^+^ in the presence or absence of 30 μM BAPTA-AM plus **b**, **c** 750 nM rapamycin or **d**, **e** 50 μM CQ for 30 h. (**b**) LC3-II levels were detected by immunoblotting with anti-LC3B antibodies. **c**, **d** MTT reduction assays were performed to assess cell viability expressed as a percentage of the untreated control cells (100%). Bars represent the mean ± SEM of three independent experiments in triplicate. ^*^p < 0.05; ^***^p < 0.001; NS, not significant. **e** After treatment with the indicated combination of drugs, MN9D cells were stained with 0.75 μM MitoTracker Red CMXRos and imaged using fluorescence microscopy. The scale bar represents 200 μm

Elevated cytosolic Ca^2+^ levels promote autophagy via the calcium/calmodulin-dependent protein kinase (CaMK)-β-AMP-activated protein kinase (AMPK)-mammalian target of rapamycin (mTOR) pathway^39^. To explore whether MPP^+^-induced cytosolic Ca^2+^ elevation regulates mTOR signaling in MN9D cells, we measured mTOR activity by checking the phosphorylation status of mTOR and p70S6K. Immunoblotting analyses showed that the levels of p-mTOR and p-p70S6K were reduced after 24 h of MPP^+^ incubation, whereas MPP^+^-induced reduction was restored in MN9D cells co-treated with MPP^+^ and BAPTA-AM (Fig. 5a). To investigate whether mTOR signaling is required for the neuroprotective effect conferred by the buffering of cytosolic Ca^2+^, MN9D cells were treated with MPP^+^ in the presence or absence of rapamycin, an mTOR inhibitor. The addition of rapamycin in MPP^+^-treated cells failed to further increase LC3-II levels (Fig. 5b, lanes 3 and 4) and had no impact on cell viability (Fig. 5c, lanes 1 and 2). In MN9D cells treated with MPP^+^ and BAPTA-AM, cell viability remained unaffected regardless of rapamycin treatment (Fig. 5c, lanes 3 and 4). Similarly, no significant reduction in cell viability was caused by rapamycin in MN9D cells overexpressing calbindin-D28K (MN9D/CB; Fig. S9a). Collectively, these data demonstrate that cytosolic Ca^2+^ elevation by MPP^+^ inactivates mTOR signaling. However, the lowering of cytosolic Ca^2+^ levels had a protective effect on MPP^+^ toxicity, which was independent of mTOR activity.

Next, we addressed whether lysosomal integrity was critical for Ca^2+^-mediated cell protection against MPP^+^-mediated neurotoxicity. Hence, we analyzed the viability of cells treated with MPP^+^ alone or in combination with BAPTA-AM, in the presence or absence of CQ. CQ did not influence the cell viability of MPP^+^-treated cells (Fig. 5d). However, CQ decreased the viability of cells co-treated with MPP^+^ and BAPTA-AM to a level similar to that of cells treated with MPP^+^ alone. Consistent with this, the protective effect of calbindin-D28K on MPP^+^ toxicity was dependent on lysosomal functionality (Fig. S9b). Mitochondrial dysfunction is one of the most characteristic features of MPP^+^-induced neurodegeneration. Mitochondrial staining with MitoTracker Red revealed that MPP^+^ treatment resulted in the loss of mitochondrial membrane potential (MMP), but not when cytosolic Ca^2+^ levels were buffered with BAPTA-AM (Fig. 5e, upper panels). Moreover, we observed that the addition of CQ disrupted MMP in cells co-treated with MPP^+^ and BAPTA-AM, suggesting that autophagy–lysosomal degradation is required for mitochondrial integrity under conditions of MPP^+^ and BAPTA-AM co-treatment (Fig. 5e, lower panels). Treatment with CQ alone did not depolarize mitochondria, suggesting that lysosomal deficits may be insufficient for mitochondrial rupture. These findings imply that under normal conditions, defective lysosomes are tolerated by cells: however, because MPP^+^ disrupts mitochondria by inhibiting the mitochondrial electron transport complex I, lysosomal activity and subsequent functional autophagic processes become crucial for preserving healthy mitochondria and maintaining cell viability in cells treated with MPP^+^ and BAPTA-AM.

### Buffering of cytosolic Ca^2+^ suppresses autophagosome accumulation and lysosomal pH neutralization in MPP^+^-treated mouse cortical neurons

**Fig. 6:**
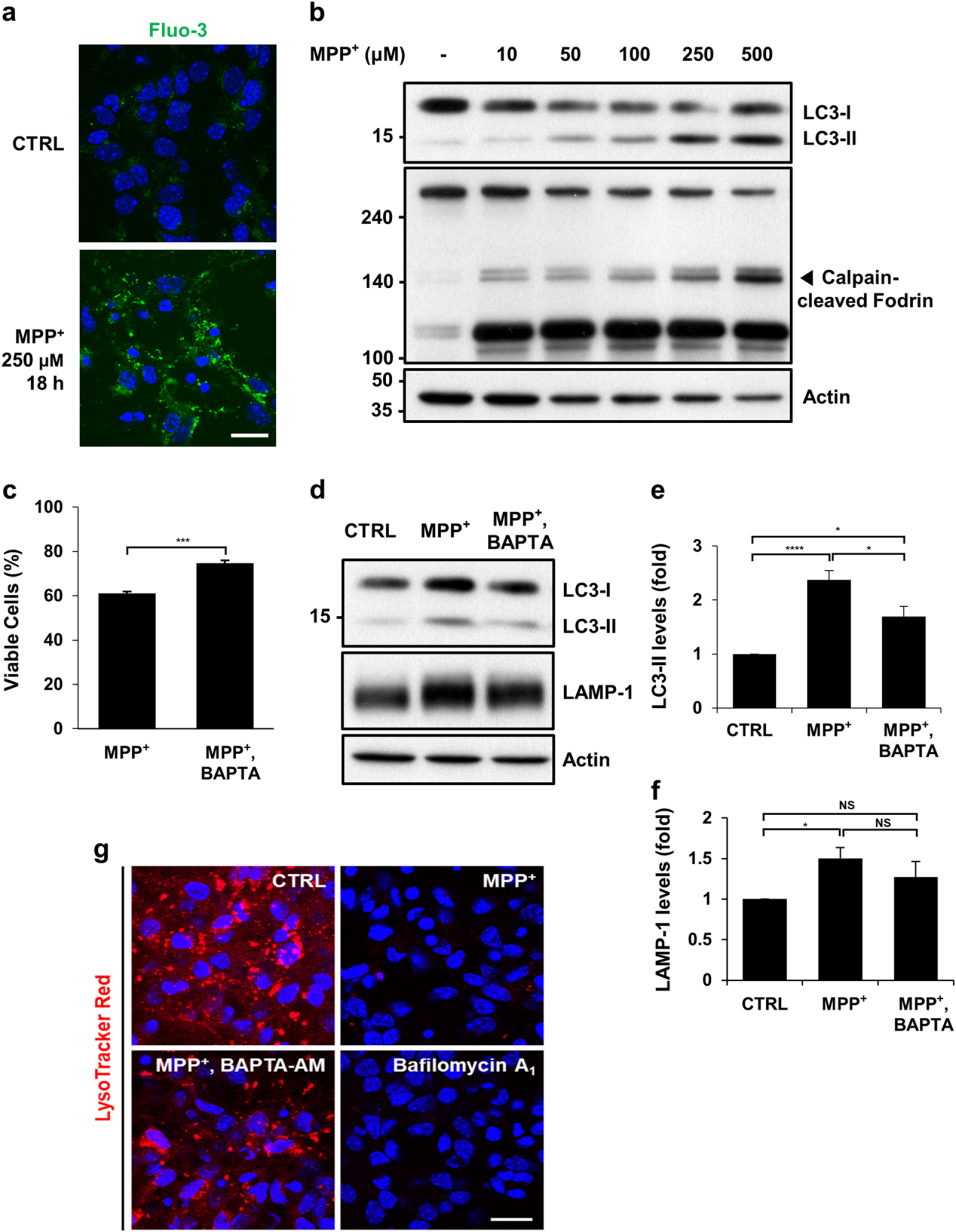
Buffering of cytosolic calcium suppresses cell death, LC3-II accumulation and lysosomal pH elevation in MPP^+^-treated cortical neurons. **a** Primary cultures of cortical neurons prepared from the cortices of E14.5 mouse embryos were treated with or without 250 μM MPP^+^ for 18 h. Cells were then loaded with 3 µM Fluo-3 and subjected to counterstaining with Hoechst dye. Fluorescent images were taken using a confocal microscopy. The scale bar represents 20 μm. **b** Cortical neurons were incubated with the indicated concentrations of MPP^+^ for 24 h. Cell lysates were subjected to immunoblot analyses using anti-LC3 or anti-fodrin antibody. Anti-actin antibody was used as a loading control. **c**–**g** Cortical neurons were treated with 250 μM MPP^+^ alone in combination with 0.5 μM BAPTA-AM for 18 h. **c** Cells were subjected to the MTT assay. Cell viability was expressed as a percentage of MPP^+^- treated cells compared to the untreated control cells (100%). Bar represents the mean ± SEM of three independent experiments in triplicate. ^***^p < 0.001. **d**–**f** Cell lysates were analyzed using immunoblotting with anti-LC3 or anti-LAMP-1 antibodies. The intensity of (**e**) LC3-II signals or (**f**) LAMP-1 from each condition was densitometrically measured using Image J. After normalization to the intensity of the actin signal, values were expressed as a fold change relative to the untreated control (value = 1). Bars represent the mean ± SEM of four independent experiments. ^*^p < 0.05; ^****^p < 0.0001; NS, not significant. **g** Cortical neurons were treated with the indicated drugs. For comparison, cells were treated with 10 nM bafilomycin A_1_ for 4 h, stained with LysoTracker Red, and counterstained with Hoechst dye. Cells were then imaged using confocal microscopy. Representative fluorescent images are provided. The scale bar represents 20 μm

To validate our data obtained from MN9D cells, primary cultures of cortical neurons were treated with MPP^+^. Fluo-3 fluorescence staining showed that MPP^+^ treatment caused a surge in cytosolic Ca^2+^ levels in cortical neurons (Fig. 6a). The calcium-activated calpain-cleaved form of fodrin was detected (Fig. 6b). Dose-dependent increase in the LC3-II form was found. Addition of BAPTA-AM inhibited MPP^+^-induced cortical neuronal death, thus confirming the neuroprotective effect of buffering of cytosolic Ca^2+^ (Fig. 6c). Similarly, BAPTA-AM inhibited MPP^+^-induced accumulation of LC3-II (Fig. 6d, e). Interestingly, levels of LAMP-1 increased in cortical neurons treated with MPP^+^ (Fig. 6d, f). Addition of BAPTA-AM reduced the MPP^+^-induced levels of LAMP-1, but these levels were still higher than those in untreated control cortical neurons. To determine lysosomal integrity in cortical neurons, lysosomal acidity was checked using the LysoTracker dye (Fig. 6g). Similar to cells treated with bafilomycin A1, cells treated with MPP^+^ almost lost the fluorescence despite containing high levels of LAMP-1, thus indicating elevated lysosomal pH. Functional lysosomes were preserved in cells co-treated with MPP^+^ and BAPTA-AM. These data suggest that cytosolic Ca^2+^ buffering protects cortical neurons from MPP^+^-mediated cytotoxicity by restoring normal lysosomal pH and autophagic flux.

## Discussion

Cytosolic Ca^2+^ is crucial for maintaining homeostasis of the nervous system by regulating neurotransmitter release and post-synaptic activity. Dysregulation of cytosolic Ca^2+^ is linked to pathological neurodegeneration^40–43^. Recent evidence implicates Ca^2+^ in the pathogenesis of PD and the regulation of Ca^2+^ may comprise a potential therapeutic target for neuroprotection in PD^44–46^. Among many hypotheses proposed to explain selective vulnerability of dopaminergic neurons in the SNpc, the maintenance of cytosolic Ca^2+^ homeostasis has drawn much attention^47^. Dopaminergic neurons in this region harbor the Ca^2+^-binding protein, calbindin-D28k that can buffer cytosolic Ca^2+,^^48^, thus substantiating the role of cytosolic Ca^2+^ buffering in PD pathogenesis. Interestingly, calbindin- D28K-positive neurons of SNpc are relatively preserved in PD postmortem samples and in monkey and mouse PD models, which were produced by the administration of the neurotoxin, MPTP^48–52^. Similarly, the membrane permeable Ca^2+^ chelator, BAPTA-AM, significantly protects cells from oxidative stress^53^. Preloading of BAPTA-AM and a calcium channel blocker suppress alpha-synuclein aggregates in HEK293T cells and SHSY-5Y cells treated with KCl^54^, supporting the notion that dysregulation of cytosolic Ca^2+^ contributes to dopaminergic neurodegeneration. Although studies involving the ectopic expression of calbindin-D28K and co-treatment with BAPTA-AM have provided additional evidence for the potential neuroprotective effects of cytosolic Ca^2+^^30–32^, the underlying mechanism remains to be determined.

We have demonstrated that MPP^+^ induces Ca^2+^-dependent cell death in the MN9D cells and primary cultures of cortical and mesencephalic neurons^22,24,55–59^. Consequently, overexpression of calbindin-D28K or co-treatment with BAPTA-AM prevents MPP^+^-induced cell death^32^. MPP^+^-induced cell death is not accompanied by biochemical and morphological features typical of apoptosis. Rather, autophagic processes is involved in MPP^+^ toxicity^25,26^. Our present study provides novel insights into the mechanism underlying the neuroprotective effects of calcium buffering in the experimental PD models. Here, we demonstrated that MPP^+^-induced autophagic alterations were a consequence of impaired autophagic degradation resulting from increased lysosomal pH. Buffering of cytosolic Ca^2+^ by BAPTA-AM co-treatment or by the overexpression of calbindin D28K preserved physiological lysosomal pH and thereby, lysosomal activity. Therefore, inhibition of a drug-induced surge of cytosolic Ca^2+^ is crucial for maintaining autophagic flux, eventually preventing MPP^+^ toxicity. Typical pathological features of PD, namely abnormal protein aggregation and mitochondrial rupture, seemed to be relieved by lowering cytosolic Ca^2+^ elevation. Taken together, our study raises the possibility that lysosomal integrity plays a crucial role in preventing MPP^+^-induced neuronal death.

Ca^2+^ is an autophagy modulator, but its effects show duality, necessitating further interpretation^60^. For example, based on the findings that inhibition of inositol 1,4,5-triphosphate receptors (IP_3_Rs) promotes autophagy^61,62^, it has been suggested that Ca^2+^ suppresses autophagy. In contrast, elevation of cytosolic Ca^2+^ stimulates autophagy via the CaMKKβ-AMPK-mTOR pathway^39^. In addition, calcineurin is required for the nuclear translocation of transcription factor EB (TFEB), triggering autophagy and lysosomal biogenesis^63^. Here, we observed that elevated cytosolic Ca^2+^ correlated with mTOR signaling in MPP^+^-treated cells, without apparent promotion of autophagy. Rather, MPP^+^-induced elevation cytosolic Ca^2+^ elevation was associated with autophagy impairment, causing lysosomal defects. In support of this argument, we demonstrated that lysosomal pH is increased by MPP^+^ in a Ca^2+^-dependent way. Lysosomal membrane permeabilization (LMP) upon MPP^+^ treatment is a cause of elevated lysosomal pH^33^. Meanwhile, cytosolic Ca^2+^ overload may lead to the continuous use of ATP by Ca^2+^ ATPases in an attempt to remove Ca^2+^ from the cytosol, thus depleting intracellular ATP. Because lysosomal acidification is conducted by v-ATPases, which pump protons into lysosomes using the energy from ATP hydrolysis, lowered ATP levels can cause incomplete acidification of the lysosomal lumen. In line with this possibility, we found that the protein levels of TFEB were diminished by MPP^+^, but not by MPP^+^ and BAPTA-AM co-treatment (unpublished data). Given that v-ATPase subunits are targets of TFEB^64^, we believe the Ca^2+^-dependent decrease in TFEB protein levels may contribute to the neutralization of lysosomal pH by influencing v-ATPases. Using the MitoTracker Red staining and the MTT reduction assays, we showed that cytosolic Ca^2+^ buffering prevents loss of MPP^+^-induced mitochondrial membrane potential and cell death, respectively. However, we found that this protective effect was abrogated when CQ inhibited lysosomal degradation (Fig. 5). Because CQ treatment does not lead to mitochondrial depolarization or decreased cell viability, lysosomal deficits may be insufficient for mitochondrial rupture or cell death. These findings imply that under normal conditions, defective lysosomes are tolerated by cells; however, lysosomal activity and functional autophagy become crucial for preserving a pool of healthy mitochondria in MPP^+^-treated cells. Under conditions in which mitochondria are damaged by treatment with MPP^+^, removing damaged mitochondria through mitophagy might be important for mitochondrial homeostasis. Therefore, the buffering of cytosolic Ca^2+^ may protect cells from MPP^+^ toxicity by rescuing the autophagy-lysosome pathway.

Among several features that contributes to selective vulnerability of dopaminergic neurons in SNpc, electrophysiological, epidemiological, and neuropathological studies have implicated that Ca^2+^ entry through Cav1 channels is amenable to phamacotherapy^46^. Consequently, accumulating evidence suggests that regulating cytosolic Ca^2+^ levels through these channels has a neuroprotective effect in animal models of PD^45,46,65^. For example, systemic administration of isradipine, a dihydropyridine antagonist of L-type Ca^2+^ channels, forces dopaminergic neurons in rodents to revert to a juvenile, Ca^2+^-independent mechanism to generate autonomous activity. More importantly, antagonist-induced reversion confers protection against dopaminergic neurotoxins. It is noteworthy that isradipine is currently being evaluated in a phase III clinical trial study for patients with early PD, showing that dysregulated Ca^2+^ homeostasis is an attractive potential target for PD drug development. Accordingly, our study may shed light on the mechanisms underlying future Ca^2+^-modulating therapies for PD, highlighting the vital role of the autophagy-lysosome pathway.

## Materials and methods

### Cell culture and drug treatment

All experimental procedures were approved by the Institutional Animal Care and Use Committee of Yonsei University (permissions: IACUC 2017-10-647-01 and 2018-01-689-01). The MN9D neuronal cell line was established by somatic fusion between embryonic mesencephalic neurons and N18TG neuroblastoma^28,29^, and cultured as previously described^25,26^. Briefly, MN9D cells were grown at 37 °C in Dulbecco’s modified Eagle’s medium (DMEM; Sigma-Aldrich, D5648) supplemented with 10% fetal bovine serum (FBS; Gibco, 26140-079) on culture dishes coated with 25 µg/ml poly-D-lysine (Sigma-Aldrich, P0899) in an atmosphere of 90% air and 10% CO_2_. MN9D cells were either left untreated or treated for the indicated time periods with 50 μM 1-methyl-4-phenylpyridinium (MPP^+^; Sigma-Aldrich, D048) alone or in combination with 30 μM 1,2-Bis(2-aminophenoxy)ethane-N,N,N′,N′-tetraacetic acid tetrakis(acetoxymethyl ester) (BAPTA-AM; Life Technologies, B6769), 50 μM chloroquine (CQ; Sigma-Aldrich, C6628), 25 nM bafilomycin A_1_ (Sigma-Aldrich, B1793), 750 nM rapamycin (Sigma-Aldrich, R-5000), or 500 nM Torin-1 (Cell Signaling Technology, 14379). Concentrations and durations of drug treatment were empirically determined. Previously established MN9D stable cell lines overexpressing calbindin-D28K^32^ were maintained in culture medium containing 250 μg/ml G418(A.G. Scientific, G1033). For preparing primary cultures of cortical neurons, cerebral cortices were removed from gestational day 14.5 mouse embryos (Orient, Gyeong-gi, Republic of Korea) and mechanically dissociated as previously described^66^. Briefly, dissociated cortical cells were plated at a density of 5 × 10^6^ cells per well of six-well plates or at 1 × 10^6^ cells per well of 24-well plates pre-coated with 100 μg/mL poly-D-lysine and 1 μg/mL laminin (Invitrogen, 23017-015). Cortical neurons were incubated at 37^o^C in MEM (Gibco, 11090-081) containing 0.6% glucose (Gibco, 15023-021), 1 mM sodium pyruvate (Sigma, P5280), 2 mM L-glutamine (Sigma, G8540), 100 units/ml penicillin-streptomycin (Thermo Fisher Scientific, 15140122), and 10% FBS in the atmosphere of 95% air and 5% CO_2._ At 24 h, culture medium was changed to Neurobasal medium (Invitrogen, 21103049) supplemented with 2% B-27 (Gibco, 17504044), 0.5 mM l-glutamine and 10 μM cytosine β-d-arabinofuranoside (Ara-C, Sigma-Aldrich, C1768). At 4 days in vitro (DIV), cultures were treated with the indicated drugs that were dissolved in the same medium.

### Measurement and imaging of intracellular Ca^2+^

Cells were stained with 3 µM Fluo-3 (Life Technology, F1242) mixed with pluronic acid (Life Technology, P3000MP) for 30 min at 37 °C and washed twice with DMEM. For flow cytometry, cells were trypsinized, and 20,000 cells per condition per experiment were analyzed using FACSCalibur and CellQuest (BD Biosciences). For obtaining fluorescent images, Fluo-3 loaded cells were mounted with Vectashield mounting medium (Vector Laboratories, H1000). Fluorescence images were acquired using a confocal microscope equipped with epifluorescence and a digital image analyzer (LSM 700, Carl Zeiss).

### Cell viability assay

Following drug treatment, the rate of cell viability was measured using the 3-(4,5-dimethylthiazol-2-yl)-2,5-diphenyltetrazolium bromide (MTT) reduction assay, as described previously^67^. Briefly, cells cultured on 24-well plates were incubated with 1 mg/ml MTT solution (Sigma-Aldrich, M2128) at 37 °C for 1 h and lysed for 18 h in an extraction buffer containing 20% sodium dodecyl sulfate (SDS) in 50% aqueous dimethylformamide. The optical densities of formazan were measured at 590 nm and 650 nm as test and reference wavelengths, respectively, using a VICTOR™ X5 Multilabel Plate Reader (PerkinElmer). Cell viability was expressed as a percentage relative to the value in untreated control (100%).

### Transmission electron microscopy

Electron microscopy was performed as previously described^27^. Briefly, MN9D cells grown in petri dishes were treated with 50 μM MPP^+^ alone or in combination with 30 μM BAPTA-AM for 30 h followed by fixation with a mixture of 2% formaldehyde and 0.2% glutaraldehyde (Polysciences, Inc., 01909) in 0.1 M cacodylate buffer (pH 7.2) for 30 min at 37 °C. Free aldehyde groups were blocked for 1 h by soaking the cells in 50 mM ammonium chloride in 0.1 M cacodylate buffer. Cells were mechanically removed, sedimented by centrifugation, enclosed in liquefied 2% agarose, and then post-fixed for 1 h with 1% osmium tetroxide (Electron Microscopy Sciences, EMS, 19152) in distilled water. This step was followed by en bloc staining with 1% aqueous uranyl acetate for 1 h. Cells were then subjected to dehydration in a graded ethanol series and embedded in Epon-Araldite (Fluka, Germany, 45345). Ultrathin sections (80 nm thickness) were prepared on cupper slot grids, stained with uranyl acetate and lead citrate, and observed at 80 kV with a Hitachi H-7650 electron microscope (Hitachi). Electron micrographs were taken with an 11-megapixel CCD XR611-M digital camera (Advanced Microscopy Techniques).

### Immunoblot analyses and immunofluorescence staining

At various times after drug treatment, cells were lysed on ice in phosphate-buffered saline (PBS; Lonza, 17-517Q) containing 1% Triton X-100 (Sigma, T8787), 1% SDS, and complete protease inhibitor cocktail (Roche, 1873580), and sonicated for homogenization. Cell lysates were centrifuged at 13,000 × *g* for 15 min at 4 °C. Supernatant proteins were collected and quantified using the Bradford protein assay reagent (Bio-Rad, 500-0006). For preparing Triton X-100 (TX)-soluble and -insoluble fraction, cells were lysed on ice in PBS containing 1% Triton X-100 and complete protease inhibitor cocktail for 30 min, and then homogenized using a 1-ml syringe with a 26-gauge needle. After centrifugation at 15,000*×g* for 30 min at 4 °C, supernatants were collected as TX-soluble fractions. After four washes with PBS containing 1% Triton X-100, the pellets were resuspended in 8 M urea buffer containing 1% SDS, 1% Triton X-100, and complete protease inhibitor, and sonicated. TX-insoluble fractions were collected by centrifugation at 15,000 × *g* for 15 min at 4 °C. Approximately 10–50 μg of protein per sample was separated by electrophoresis on 8–15% SDS-polyacrylamide gels and transferred to polyvinylidene fluoride membranes (Pall Corp., 66543). Membranes were probed with primary antibodies overnight at 4 °C and washed with Tris-buffered saline containing 0.1% Tween-20 (TBST). The following primary antibodies were used: rabbit anti-LC3 (Cell Signaling Technology, 2775), guinea pig anti-p62 (Progen, GP62-C), mouse anti-ubiquitin (P4D1, Santa Cruz, SC-8017), rat anti-LAMP-1 (Developmental Studies Hybridoma Bank, 1D4B), rabbit anti-rab5 (Cell Signaling, 2143), rabbit anti-rab7 (Cell Signaling Technologies, 9367), rabbit anti-p-mTOR (S2448; Cell Signaling Technology, 2971), rabbit anti-mTOR (Cell Signaling Technology, 2972), rabbit anti-p-p70S6K (T389; Cell Signaling Technology, 9234), rabbit anti-p70S6K (Cell Signaling Technology, 2708), mouse anti-fodrin antibody (ENZO Life Sciences, BML-FG6090), rabbit anti-calbindin-D28K (Swant, 300), mouse anti-GAPDH (EMD Millipore, mab374), and rabbit anti-actin antibody(Sigma-Aldrich, A2066). After extensive washes with TBST, blots were incubated with the appropriate horseradish peroxidase (HRP)-conjugated secondary antibodies for 1 h at room temperature. The secondary antibodies used included HRP-conjugated anti-rabbit (Santa Cruz, sc-2004), HRP-conjugated anti-mouse (Santa Cruz, sc-2005), HRP-conjugated anti-guinea pig (Sigma-Aldrich, A5545), and HRP-conjugated anti-rat antibody (Santa Cruz, sc-2006). Specific bands were visualized using an enhanced chemiluminescence kit (ECL; PerkinElmer Waltham, NEL105). The relative intensity of each band was measured using ImageJ Imaging Software (National Institute of Health, Bethesda, MD). For immunofluorescence staining, cells were grown on coverslips, and treated, fixed with 4% paraformaldehyde (EMS, 15170) at room temperature for 15 min, and permeabilized with 0.1% saponin (Sigma, S4521) for 10 min. Coverslips were washed and incubated in PBS containing 0.2% Triton X-100 and 5% normal goat serum (Invitrogen, 16210) for 1 h to block nonspecific sites. Subsequently, cells were incubated overnight at 4 °C with primary antibody in PBS containing 0.2% Triton X-100 and 1% normal goat serum. After washing with PBS, cells were incubated at room temperature for 1 h with the appropriate secondary antibody. These included Alexa488-conjugated goat anti-guinea pig IgG (Invitrogen, A11073), Alexa 488-conjugated goat anti-rabbit IgG (Invitrogen, A11008), Alexa 568-conjugated goat anti-rabbit IgG (Invitrogen, A11011), and Alexa 568-conjugated goat anti-mouse IgG (Invitrogen, A11004). For counterstaining nuclei, 1 μg/ml Hoechst 33258 (Molecular Probes, H-1398) was used. Cells were then mounted with Vectashield (Vector Laboratories, H1000). Fluorescent images were acquired using a confocal laser scanning microscope (Zeiss LSM 700). As previously described^25,26^, acquired images were analyzed for LC3 puncta using ImageJ Imaging Software.

### Analyses of lysosomal acidification and mitochondrial membrane potential

Following drug treatment, cells were incubated with 0.5 μM LysoTracker Red DND-99 (Life Technologies, L7528) or 0.75 μM MitoTracker Red CMXRos (Life Technologies, M7512) for 30 min at 37 °C. Subsequently, cells were washed twice with PBS. Fluorescent images of live cells were observed under an Axio Observer A1 microscope (Carl Zeiss). Cathepsin B activity was examined using the Magic Red Cathepsin B detection kit (ImmunoChemistry Technologies, 937). Briefly, Magic Red Cathepsin B reagent was added to the cell medium, and after 1 h, cells were washed twice with PBS. Fluorescent images were taken using an Axio Observer A1 microscope. For monitoring autophagic flux, MN9D cells plated on poly-D-lysine-coated culture dishes were cultivated for 2 days and subjected to transient transfection with an mRFP-EGFP tandem fluorescent-tagged LC3 probe (a generous gift from Prof. Tamotsu Yoshimori at Osaka University, Japan) for 24 h using Lipofectamine 2000 (Thermo Fisher Scientific, 11668019) as recommended by the supplier. Drug treatment was performed 24 h post-transfection.

### Statistics

Data were expressed as means ± standard error of the mean (SEM). from at least three independent experiments. The differences were determined by one-way ANOVA or two-tailed unpaired *t*-test using GraphPad Prism 5. Values of ^****^p < 0.0001, ^***^p < 0.001, ^**^p < 0.005, or ^*^p < 0.05 were considered statistically significant.

## Supplementary information

Supplementary figure 1

Supplementary figure 2

Supplementary figure 3

Supplementary figure 4

Supplementary figure 5

Supplementary figure 6

Supplementary figure 7

Supplementary figure 8

Supplementary figure 9
