# Supplementary figures and images for: Buffering of cytosolic calcium plays a neuroprotective role by preserving the autophagy-lysosome pathway during MPP+-induced neuronal death
Source: Cell Death Discov. 2019 Aug 19;5:130. doi: 10.1038/s41420-019-0210-6 (PMC6700189; doi:10.1038/s41420-019-0210-6)

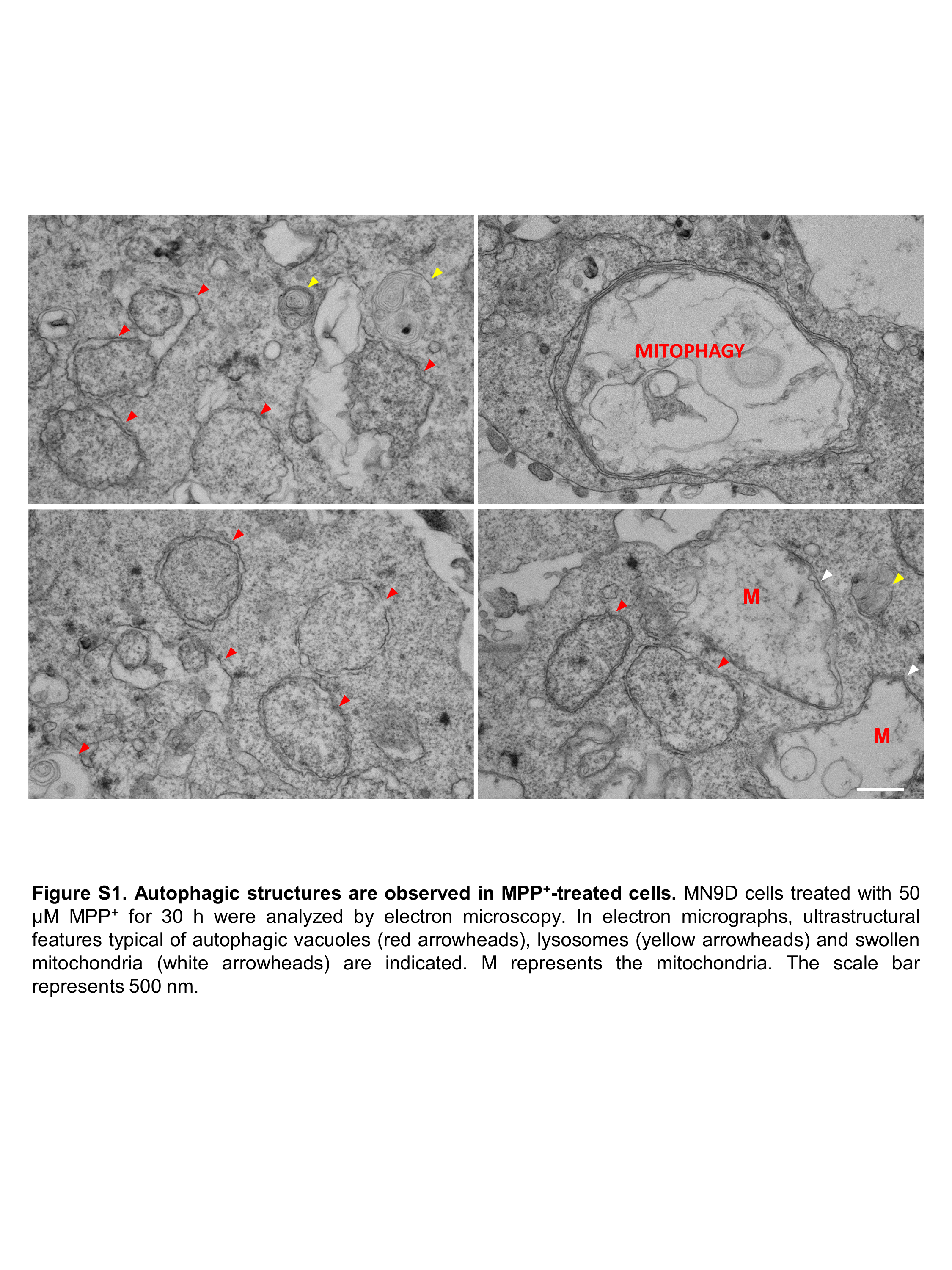

Supplement: Supplementary file 1 — Supplementary figure 1 [file 41420_2019_210_MOESM1_ESM.tif]

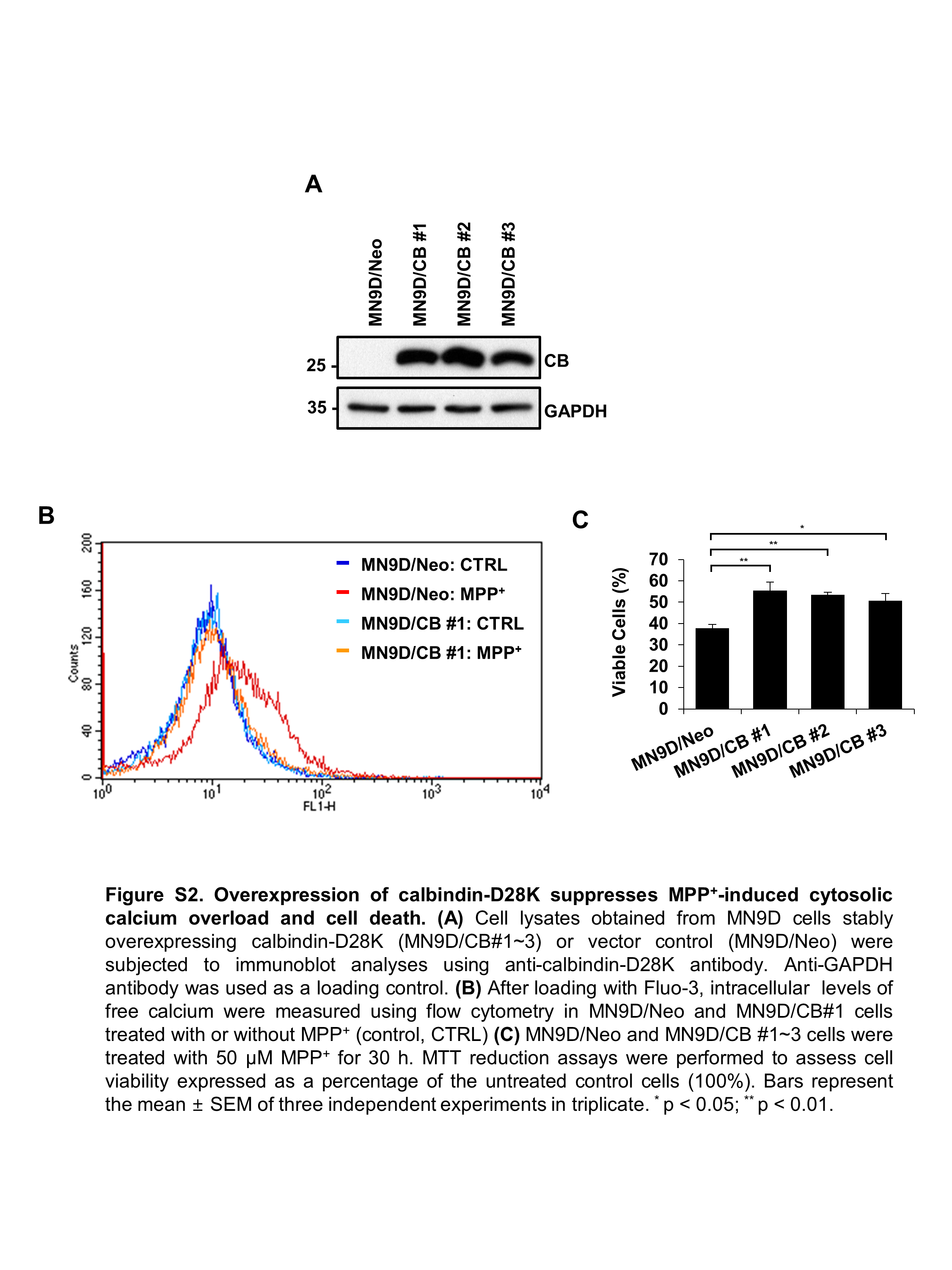

Supplement: Supplementary file 2 — Supplementary figure 2 [file 41420_2019_210_MOESM2_ESM.tif]

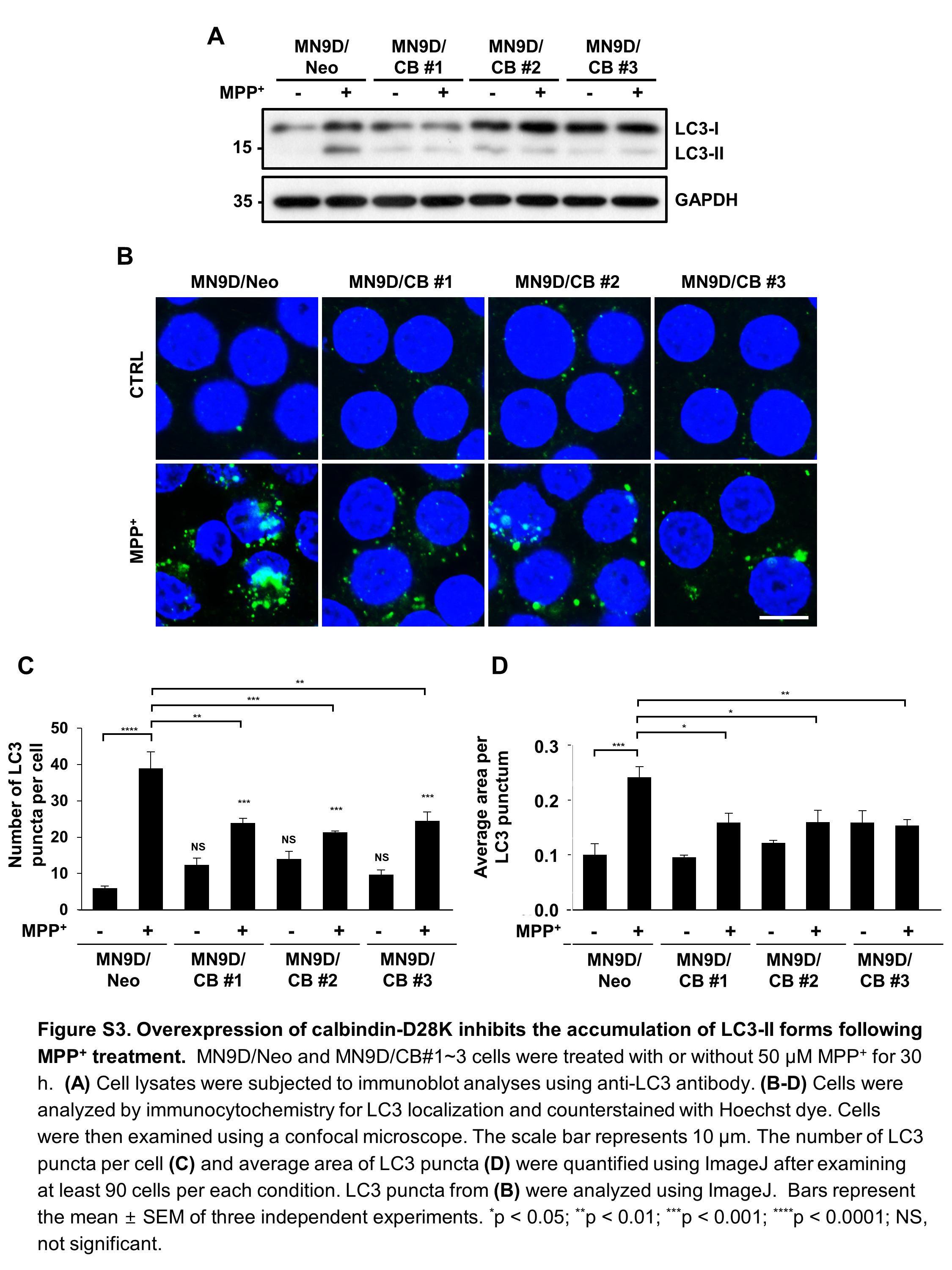

Supplement: Supplementary file 3 — Supplementary figure 3 [file 41420_2019_210_MOESM3_ESM.tif]

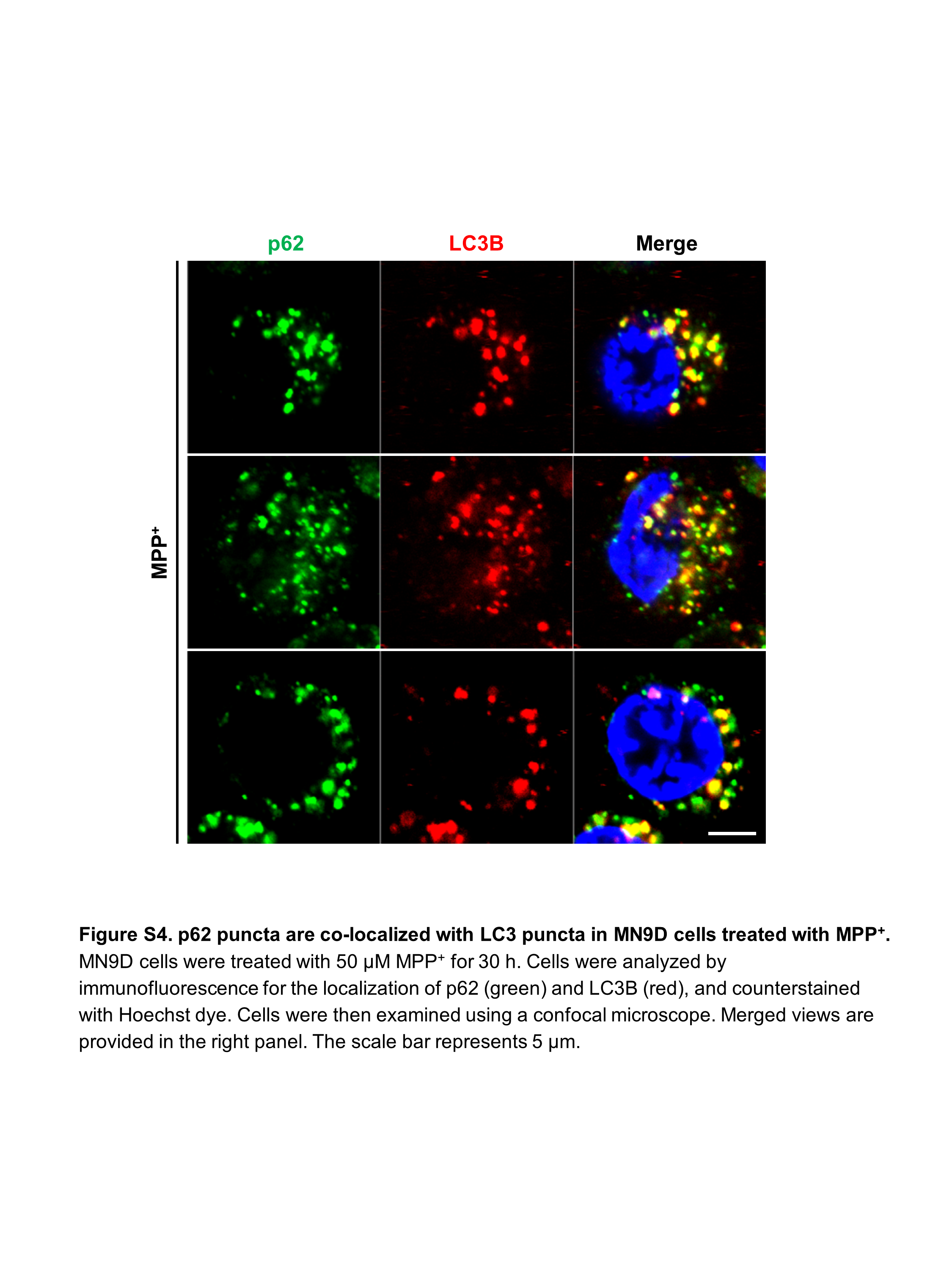

Supplement: Supplementary file 4 — Supplementary figure 4 [file 41420_2019_210_MOESM4_ESM.tif]

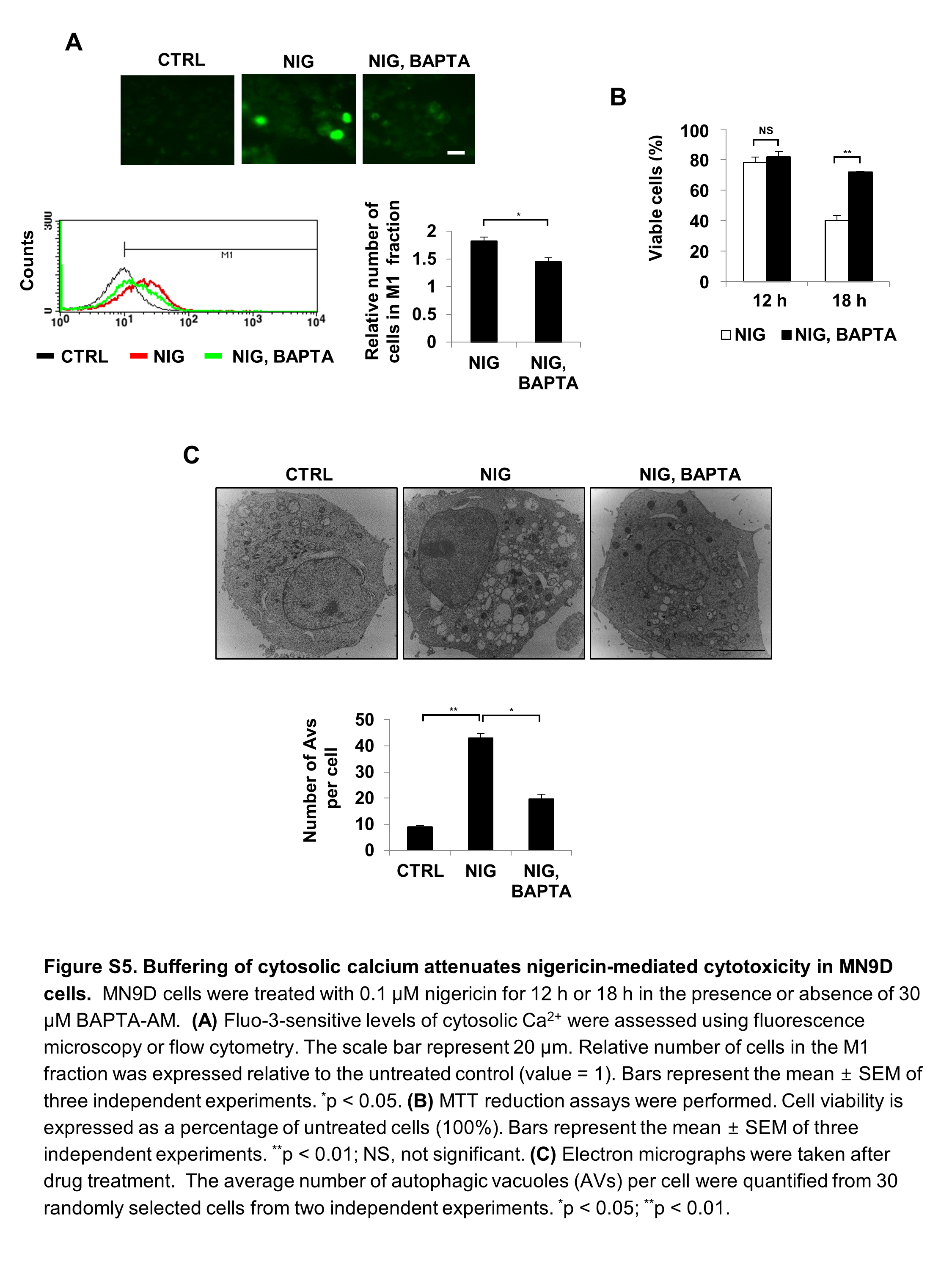

Supplement: Supplementary file 5 — Supplementary figure 5 [file 41420_2019_210_MOESM5_ESM.tif]

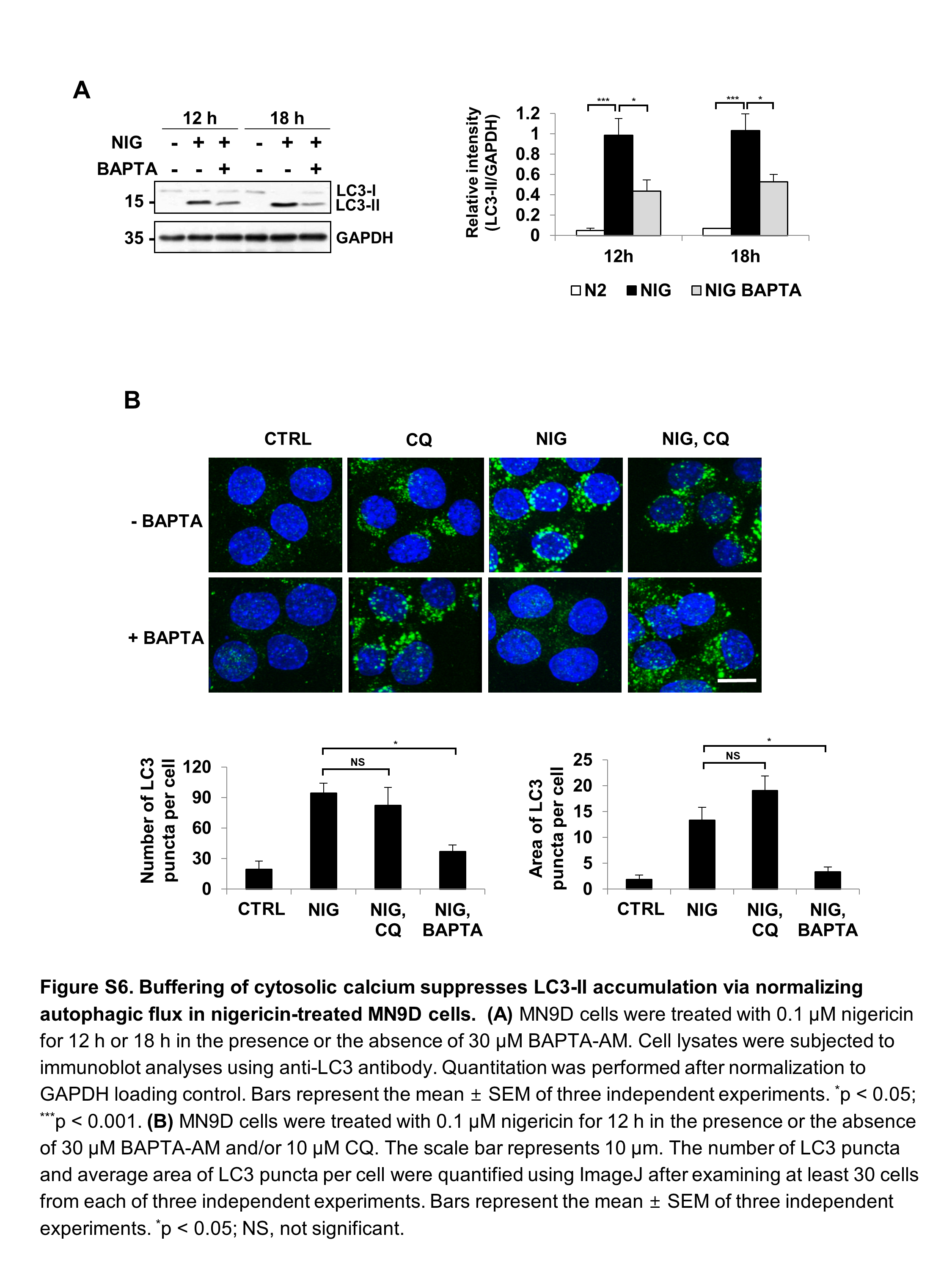

Supplement: Supplementary file 6 — Supplementary figure 6 [file 41420_2019_210_MOESM6_ESM.tif]

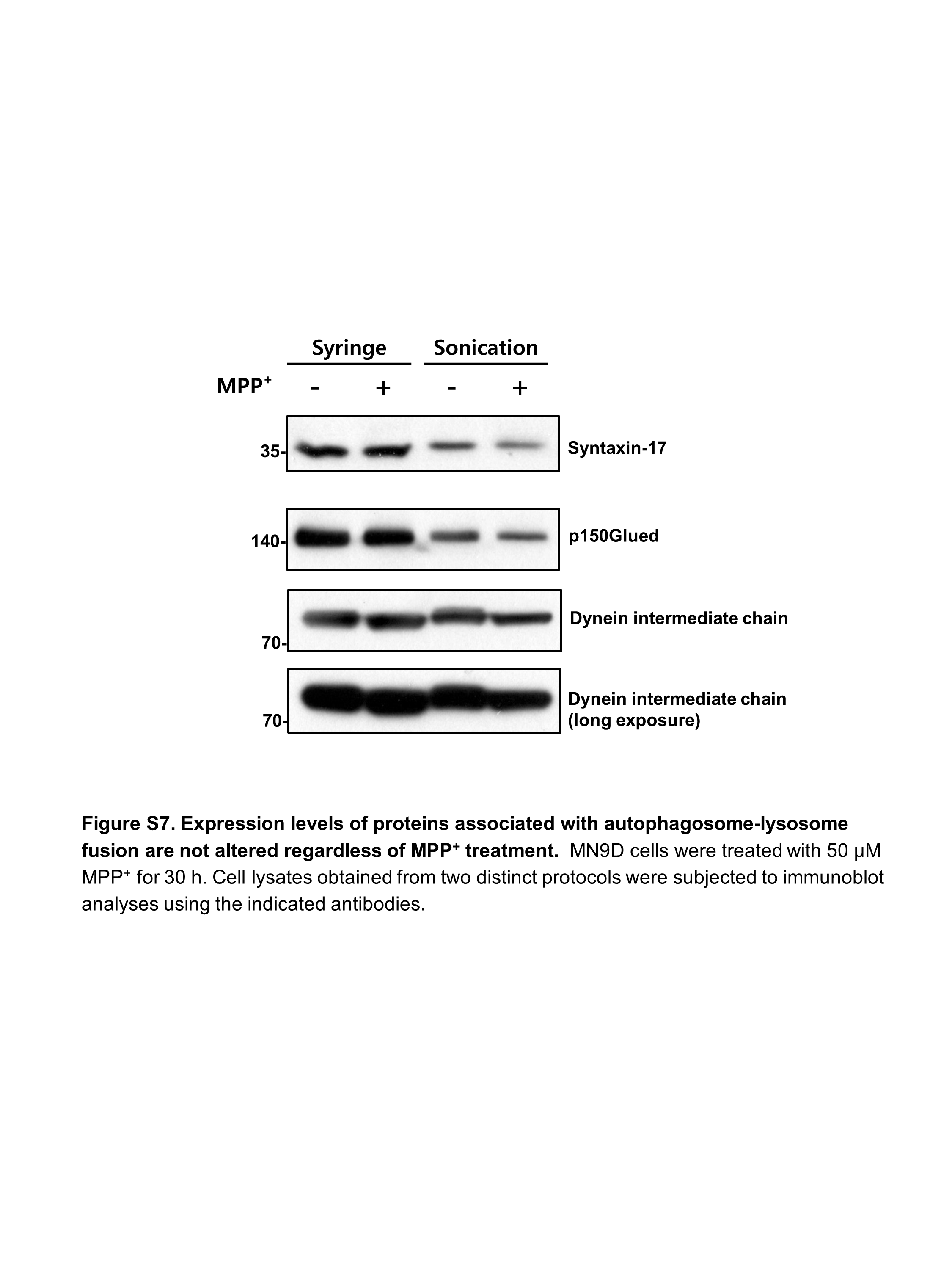

Supplement: Supplementary file 7 — Supplementary figure 7 [file 41420_2019_210_MOESM7_ESM.tif]

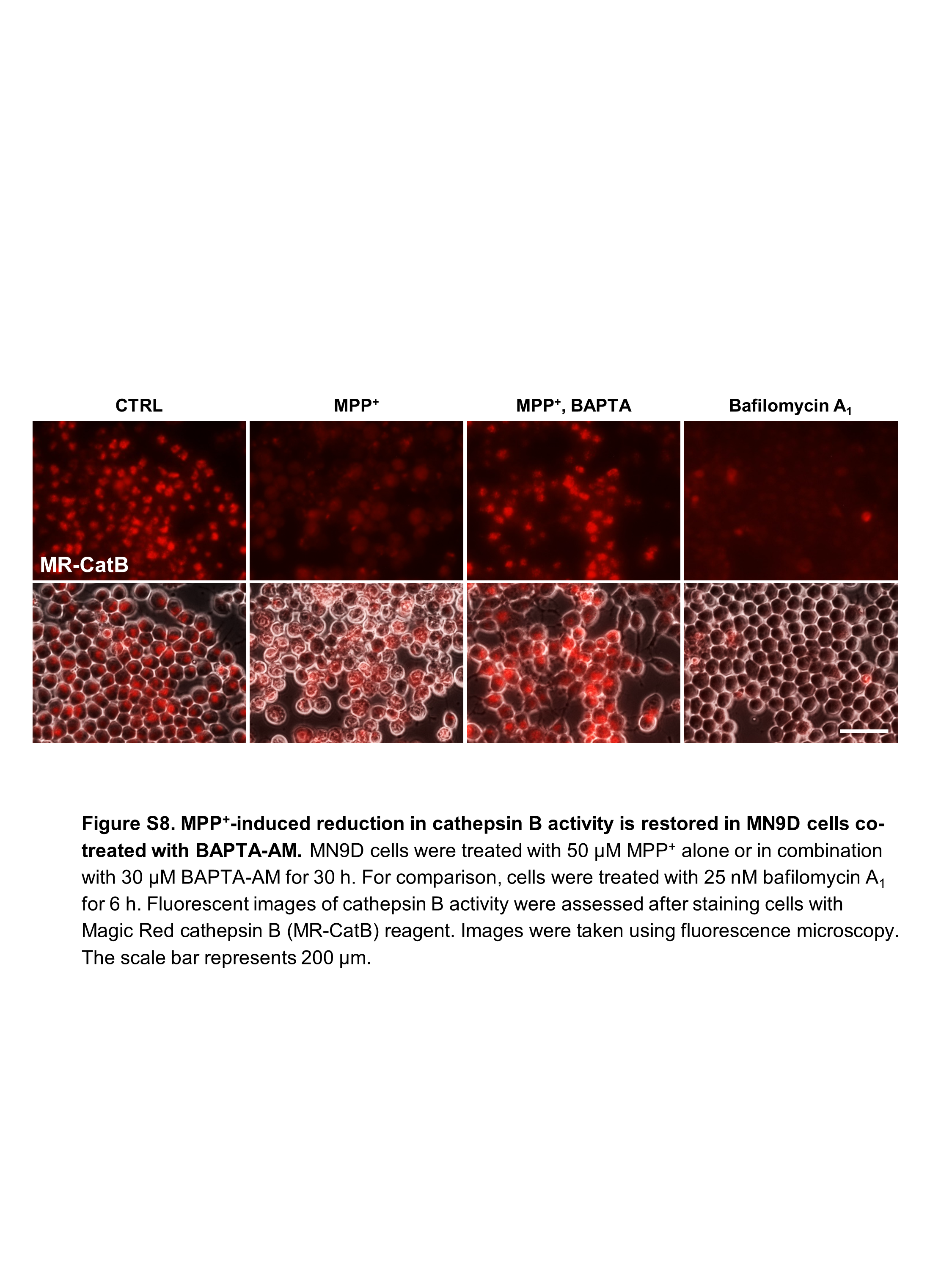

Supplement: Supplementary file 8 — Supplementary figure 8 [file 41420_2019_210_MOESM8_ESM.tif]

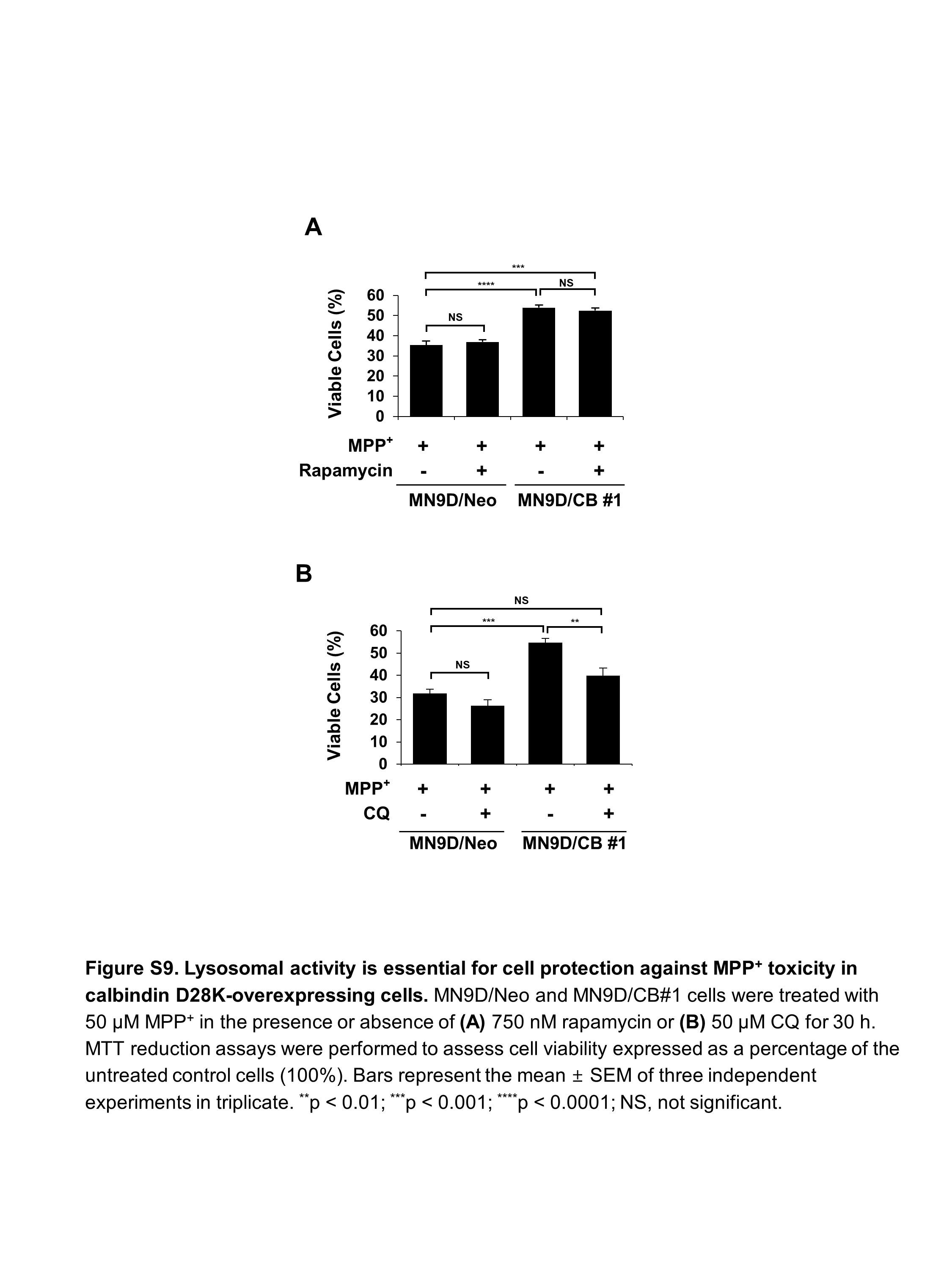

Supplement: Supplementary file 9 — Supplementary figure 9 [file 41420_2019_210_MOESM9_ESM.tif]
